# The Reliability of Measuring Muscle Cross‐Sectional Area in Children Undergoing Treatment for Musculoskeletal Sarcoma

**DOI:** 10.1002/jcsm.70231

**Published:** 2026-02-12

**Authors:** Claire Laurie, Leo Donnan, Catherine L. Granger, Alicia J. Spittle, David D. Eisenstat, Timothy Cain, Nisarg Mehta, Christopher Godfrey, Tara L. FitzGerald

**Affiliations:** ^1^ Department of Physiotherapy The University of Melbourne Melbourne Victoria Australia; ^2^ Murdoch Children's Research Institute Melbourne Victoria Australia; ^3^ The Royal Children's Hospital Melbourne Victoria Australia; ^4^ The Royal Women's Hospital Melbourne Victoria Australia; ^5^ Department of Paediatrics The University of Melbourne Melbourne Victoria Australia; ^6^ The Royal Melbourne Hospital Melbourne Victoria Australia; ^7^ Alder Hey Children's Hospital Liverpool UK

**Keywords:** cancer, magnetic resonance imaging, paediatric oncology, reliability, sarcoma, sarcopenia, skeletal muscle

## Abstract

**Background:**

Changes in musculoskeletal health and function are increasingly recognised as important long‐term consequences of treatment for malignant bone tumours in paediatric populations. Accurate and reliable assessment methods are critical for monitoring these changes during treatment. We aimed to determine the inter‐ and intra‐rater reliability of MRI‐based measurements of muscle cross‐sectional area in children undergoing treatment for paediatric musculoskeletal sarcoma.

**Methods:**

We conducted a retrospective cohort study at a tertiary paediatric cancer centre. Eligible participants were aged 2–17 years and had undergone routine clinical MRI scans as part of their treatment for musculoskeletal sarcoma. Muscle cross‐sectional area was measured, focusing on key lower limb muscle/muscle groups. Three trained raters independently performed all measurements. Inter‐ and intra‐rater reliability were assessed using intraclass correlation coefficients (ICCs) with 95% confidence intervals (CIs). Measurement precision was evaluated using minimal detectable change (MDC) values, expressed as a percentage of mean muscle size.

**Results:**

Nineteen patients (mean age 11.6 ± 2.6 years; 11 female and 8 male) were included. Diagnoses included osteosarcoma (*n* = 12), Ewing sarcoma (*n* = 5), rhabdomyosarcoma (*n* = 1) and synovial sarcoma (*n* = 1). The most common tumour location was the distal femur (*n* = 9), followed by proximal tibia (*n* = 2), pelvis (*n* = 2), proximal fibula (*n* = 1), distal fibula (*n* = 1) and other sites (*n* = 4). Metastatic disease was present in seven patients, while 12 had localised disease. Once images affected by tumour were excluded, a minimum of 60 images of each muscle/muscle group were included for analysis.

Inter‐rater reliability was excellent for psoas (ICC = 0.97, 95% CI: 0.95–0.98), gracilis (ICC = 0.96, CI: 0.93–0.98), medial gastrocnemius (ICC = 0.91, CI: 0.86–0.94), mid‐femoral muscle circumference (ICC = 0.99, CI: 0.99–0.99) and mid‐tibial muscle circumference (ICC = 0.99, CI: 0.99–0.99). Good inter‐rater reliability was found for rectus femoris (ICC = 0.88, CI: 0.83–0.95) and biceps femoris (ICC = 0.83, CI: 0.75–0.89). Intra‐rater reliability was excellent across all muscles assessed (ICCs: 0.92–0.99).

MDC values indicated highest measurement precision for mid‐femoral muscle circumference (10.62%), mid‐tibial muscle circumference (11.69%) and psoas (18.26%), enabling detection of clinically meaningful changes over time.

**Conclusions:**

This study demonstrates that MRI measurement of muscle cross‐sectional area in children with musculoskeletal sarcoma is a reliable tool. MDC values allow for identification of true muscle loss, supporting early intervention.

**Abbreviations**
BF: biceps femoris muscle
CI: confidence interval
CT: computed tomography
EMR: electronic medical record
G: gracilis muscle
ICC: intraclass correlation coefficient
MDC: minimal detectable change
MFC: mid‐femoral muscle circumference
MHG: medial head of gastrocnemius muscle
MRI: magnetic resonance imaging
MTC: mid‐tibial muscle circumference
P: psoas muscle
PACS: Picture Archiving and Communication System
PET: positron emission tomography
PET‐MRI: positron emission tomography combined with whole‐body magnetic resonance imaging
RF: rectus femoris muscle
ROI: region of interest

## 1 Introduction

Survival rates for paediatric malignant bone tumours are improving globally [1, 2]. As survival rates rise, it is crucial to recognise the long‐term consequences of treatment, which include significant changes in musculoskeletal health and function [3, 4]. Osteosarcoma, Ewings sarcoma, rhabdomyosarcoma and synovial sarcoma (referred to in this study as musculoskeletal sarcoma) represent around 10% of all paediatric solid tumours [5]. Typically, after a diagnostic biopsy and disease staging, treatment involves a period of intensive chemotherapy followed by biological reconstruction, endoprosthetic reconstruction, rotationplasty or amputation of the affected limb, and may also include radiation to the involved field for local control. The recovery from this surgery can take months to years [1, 6]. Unfortunately, children who have been treated for musculoskeletal sarcoma often have long‐term changes in the way they move and walk, affecting their health‐related quality of life [6, 7].

Sarcopenia, which refers to the reduction in the quantity, quality and function of skeletal muscle and its measurement, has emerged as a valuable quantitative tool for assessing changes associated with various disease processes, including cancer [8, 9]. Challenges remain in the methodological approach, particularly in paediatric populations [10, 11]. It is well established that chemotherapy has a detrimental effect on skeletal muscle [12]. Recent studies indicate that changes in muscle mass are linked to functional outcomes in children with leukaemia and neuroblastoma [13, 14, 15]. Although it has yet to be explored, this effect is likely to be particularly significant for children with musculoskeletal sarcoma, as any changes in musculoskeletal function in early treatment is likely to have a negative effect on the rehabilitation or postsurgical stage of their care. Considering the complex nature of treating musculoskeletal sarcoma and the demanding rehabilitation processes involved, it is essential to identify key muscles/muscle groups and comprehend the alterations in muscle properties and physical function that transpire during the presurgery phase of treatment. This understanding is a vital step in identifying these losses and optimising functional recovery postsurgery.

Determining the mass of a skeletal muscle can be accomplished using many different methods [16, 17]. Sequential magnetic resonance imaging (MRI) or computed tomography (CT) images across the length of the muscle are gaining popularity [18]. However, more recently, techniques such as anthropometry and ultrasound have been explored [19]. Children undergoing treatment for musculoskeletal sarcoma undergo regular CT or MRI imaging as part of their monitoring, often accompanied by combined positron emission tomography (PET) imaging to assess treatment response and/or disease activity. Therefore, utilising these methods for assessment of muscle mass would be feasible without adding additional scans. Single‐slice MRI cross‐sectional area has been shown to be a valid method of estimating total muscle volume in the thigh and calf muscles in young adults [20, 21] and the psoas muscle in healthy children [22]. The reliability of this technique has not yet been established in children with musculoskeletal sarcoma.

The primary objectives of this study were to (1) develop a simple method using MRI to evaluate the cross‐sectional area of key muscles in children with musculoskeletal sarcoma from their respective MRI scans and (2) evaluate the inter‐ and intra‐rater reliability of this measurement tool.

## 2 Methods

### 2.1 Study Design

A single‐site retrospective cohort study was conducted at the Royal Children's Hospital in Melbourne, Australia. Positron emission tomography combined with whole‐body magnetic resonance imaging (PET‐MRI) images obtained at the time of diagnosis and 3 months later in children undergoing treatment for musculoskeletal sarcoma were analysed.

### 2.2 Setting

The study was approved by The Royal Children's Hospital Melbourne Human Research Ethics Committee—HREC/100769/RCHM‐2023.

### 2.3 Participants

Children aged 2–17 years diagnosed with musculoskeletal sarcoma between January 2016 and December 2023 were eligible according to the criteria in Table 1.

**TABLE 1:** Eligibility criteria.

Inclusion	Exclusion
Children aged 2–17 years old	Surgery completed upfront prior to any chemotherapy
Diagnosed with a musculoskeletal sarcoma (Ewing sarcoma, osteosarcoma, synovial sarcoma or rhabdomyosarcoma) of the lower limb or pelvis between January 2016 and December 2023	Disease progression on treatment leading to expedited surgery
Treated at The Royal Children's Hospital, Melbourne	

Children undergoing treatment for musculoskeletal sarcoma receive a PET‐MRI scan at diagnosis and again approximately 3 months later to assess response to initial chemotherapy and resectability of tumour prior to any surgical treatment. Assessment of imaging conducted at diagnosis and post‐initial chemotherapy has been included in this study.

### 2.4 Sampling

Children diagnosed with musculoskeletal sarcoma were identified using the electronic medical record (EMR) diagnosis coding and screened according to the eligibility criteria (Table 1).

To achieve a balance between statistical power and practical feasibility, it was determined that a sample size of 60 images of each muscle would be sufficient to provide an accurate representation of the reliability of this measurement. This is in accordance with similar studies that have assessed the reliability of MRI measurements [23, 24].

Since children receive two PET‐MRI scans during this initial phase of their treatment, and measurements can be taken from both sides of the body, 19 children were randomly selected via random number generation for inclusion to give a minimum of 60 images of each muscle, accounting for the exclusion of any image that was affected by tumour.

### 2.5 Demographics

Age at diagnosis, cancer type, tumour location, presence of metastatic disease and clinical staging were recorded for each patient.

### 2.6 Assessors

Three assessors from a range of professional backgrounds and experiences were selected to represent generalisability to clinical settings. Assessors included a specialist paediatric physiotherapist, a medical trainee in the final year of training and a senior paediatric orthopaedic fellow. Although all three assessors were clinical professionals, none had significant experience with MRI muscle measurements. Two training sessions were conducted by a paediatric radiologist. Training included (1) familiarisation with measurement protocols, (2) observation of each measurement conducted by the radiologist and (3) practice measurements with real‐time feedback and discussion.

### 2.7 MRI Image Analysis

Whole‐body PET‐MRI imaging was performed on a Siemens Healthineers biograph system. These studies were obtained as part of routine staging and treatment response assessment at diagnosis and after 3 months of chemotherapy; standard imaging included whole‐body coronal T2 STIR and whole‐body axial T2 half‐Fourier acquired single‐shot turbo spin echo (HASTE) sequences, which were used preferentially for analysis. Images were accessed from the hospital's Fujifilm Synapse Picture Archiving and Communication System (PACS). Coronal and cross‐sectional images were viewed, allowing the identification of standardised points for analysis.

**FIGURE 1:**
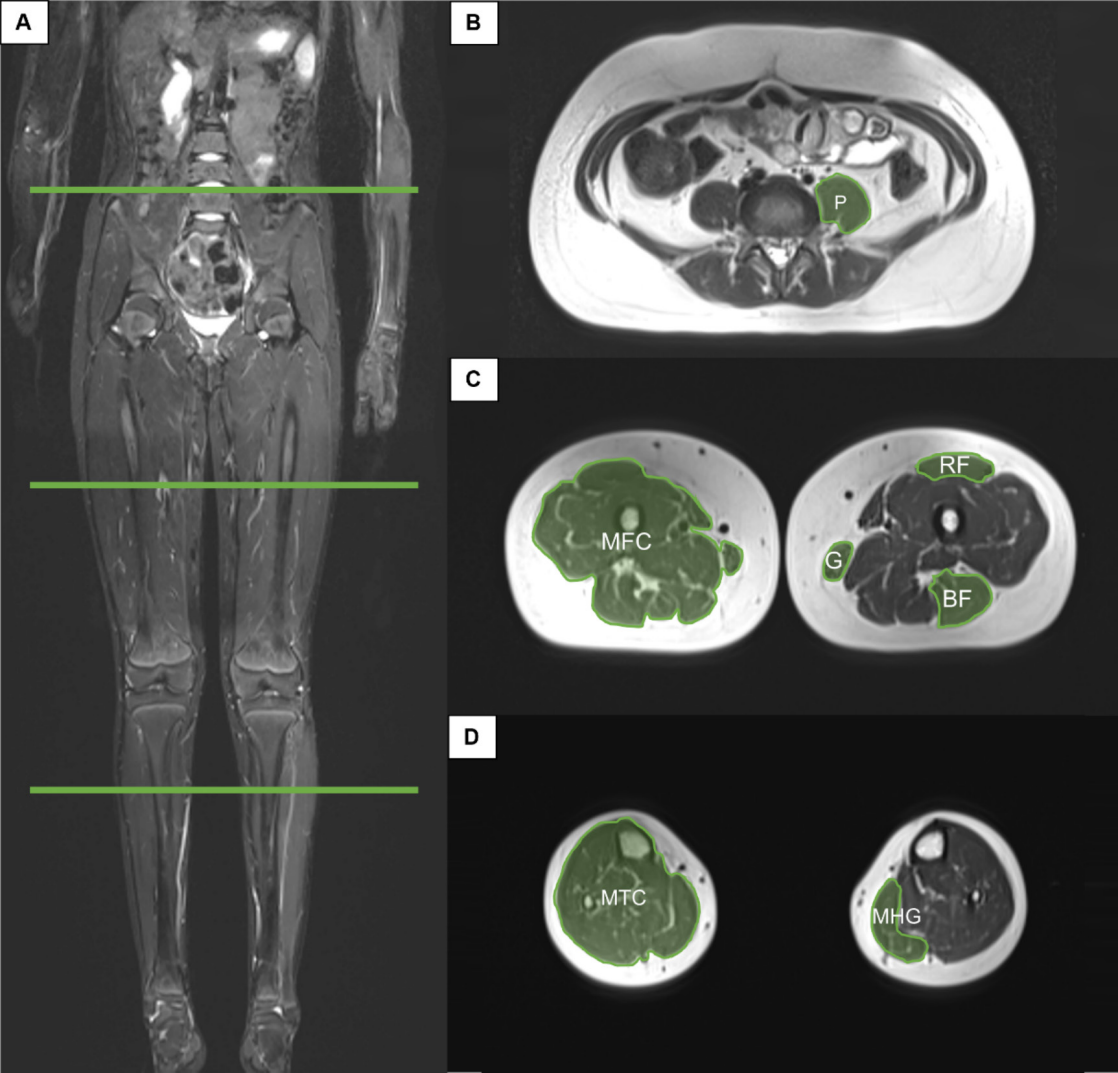
MRI measurements of muscle cross‐sectional area showing (A) coronal view with level for segmentation identified (L3/4 intervertebral disc, 50% length of the femur and 33% length of the tibia). (B) Axial view of psoas muscle; P, Psoas muscle. (C) Axial view of thigh muscles; B, Biceps femoris; G, Gracilis; MFC, Mid‐femoral muscle circumference; RF, Rectus femoris. (D) Axial view of calf muscles; MHG, Medial head of gastrocnemius; MTC, Mid‐tibial muscle circumference.

Using a combination of muscles that had been identified in previous studies, and the expertise of the authors of this study, five individual muscles and two muscle groups were identified as warranting evaluation (Figure 1). Utilising the method described by Lurz and colleagues [22], the psoas muscle (P) was evaluated at the level of the intervertebral disc between the third and fourth lumbar vertebral segments (L3/4). The rectus femoris (RF), gracilis (G) and biceps femoris muscles (BF) were each assessed at the midpoint of the femur, defined as 50% of the length measured from the most superior surface of the femoral head to the most inferior surface of the medial femoral condyle. The medial head of the gastrocnemius muscle (MHG) was assessed at 33% of the tibial length. Additionally, cross‐sectional areas of two composite muscle groups were analysed. Mid‐femoral muscle circumference (MFC) area was measured at 50% of the femur length, and mid‐tibial muscle circumference (MTC) area was measured at 33% of the tibial length. In both cases, subcutaneous fat was excluded from the measurement.

A single clinician isolated and labelled the image at the level listed above and saved the de‐identified cross‐sectional image in a private subfolder for analysis. Any muscle measurements affected by the tumour were excluded. Each examiner then opened the cross‐sectional image and used the freehand region of interest (ROI) tool to draw the circumference of the muscle. The ROI feature is part of the standard image analysis toolkit available in most commercial PACS platforms. This tool allows the user to manually delineate an arbitrary, nongeometric shape directly on the image by tracing a closed contour with a mouse or stylus. Once the loop is completed, the PACS software automatically computes quantitative metrics within the selected region, including area in square millimetres (mm^2^). Muscle images were assessed independently by each of the investigators. Investigator 1 then repeated the measurements 2 weeks later. Images were de‐identified, with no clinical information visible. Each investigator entered their measurement results into the REDCap (Research Electronic Data Capture) tool hosted at the Murdoch Children's Research Institute. REDCap is a secure, web‐based software platform designed to support data capture for research studies [25].

### 2.8 Statistical Analysis

Baseline physical characteristics were tabulated for all participants.

Measurements were exported from REDCap, and statistical analysis was performed using the Stata software (Release 18, StataCorp LLC, College Station, TX). Inter‐ and intra‐rater reliability was reported using the individual intraclass correlation coefficient (ICC) and 95% confidence interval (CI). Excellent reliability was described as greater than 0.90 and good reliability between 0.75 and 0.90 [26]. This is informed by previous studies of reliability, which found a reliability of 0.99 (95% CI 0.986 to 0.995) for the measurement of the psoas muscle in 53 normal children [16].

Minimal detectable change (MDC) was calculated for each of the muscle/muscle groups to assess the reliability of MRI measurements in this study. MDC is derived from the standard error of the measurement and therefore represents the smallest change in measurement that can be considered beyond the threshold of measurement error, ensuring that any observed change is statistically significant. This measure was used to evaluate the precision and sensitivity of the MRI technique in capturing reliable and meaningful changes over time [27].

## 3 Results

### 3.1 Participants

**FIGURE 2:**
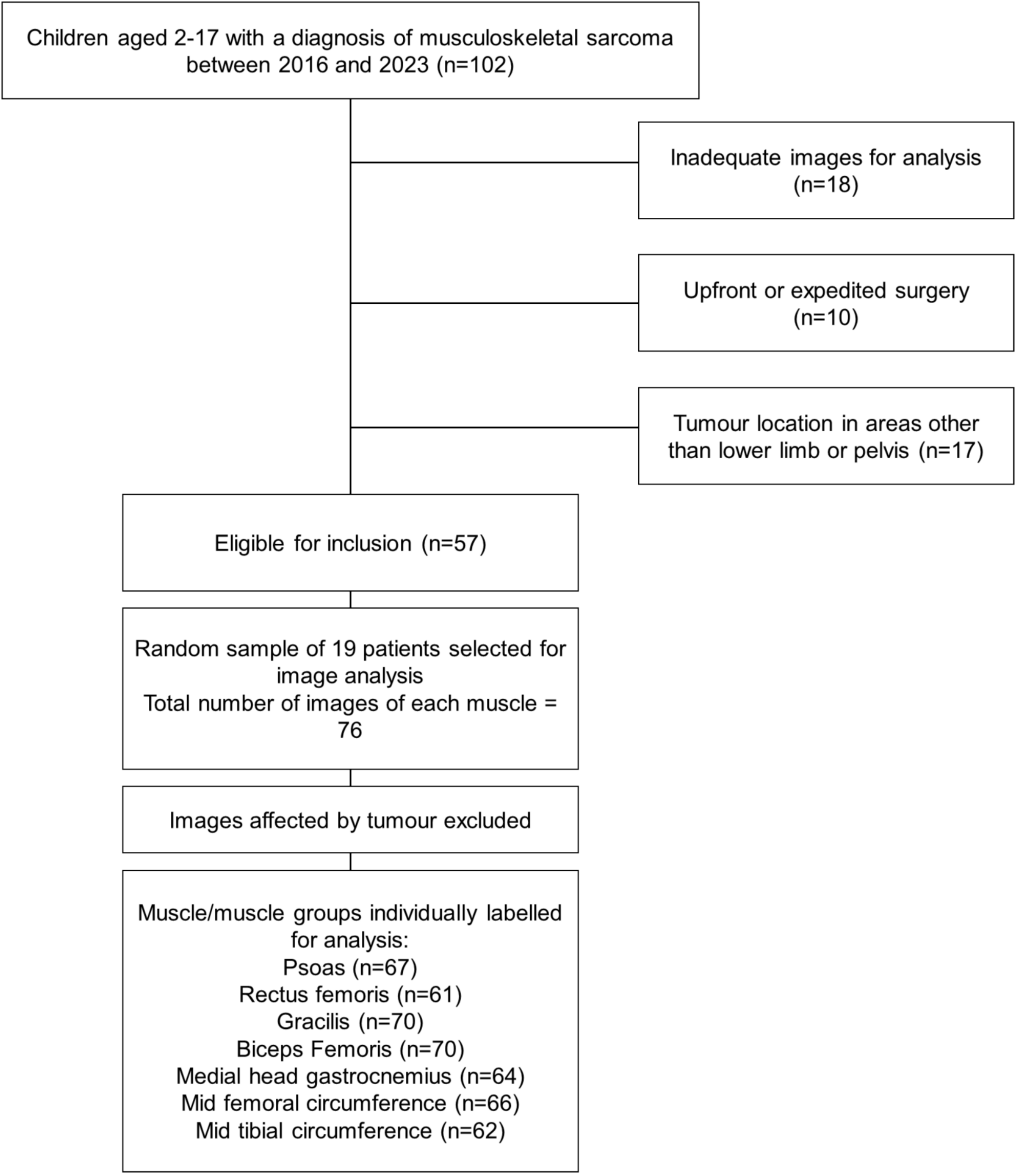
Study flow diagram.

Following the screening utilising the EMR, 57 children met the inclusion criteria (Figure 2) of which 19 children were randomly selected via a number generator for image analysis. After excluding any images affected by the tumour, a minimum of 60 images of each muscle/muscle group were included for analysis. Patient details are described in Table 2. Eight children were male, and 11 were female. The age range was between 7 and 16 years. Osteosarcoma was the most common cancer type (*n* = 12), followed by Ewings sarcoma (*n* = 5). The most common tumour locations were the distal femur (*n* = 9), pelvis (*n* = 2) and proximal tibia (*n* = 2).

**TABLE 2:** Patient characteristics (*n* = 19).

Sex	*n* (%)
Male	8 (42.1)
Female	11 (57.9)
Age	Mean (SD)
Age (mean + SD)	11.6 ± 2.6 (years)
Disease	*n* (%)
Osteosarcoma	12 (63.2)
Ewings sarcoma	5 (26.6)
Rhabdomyosarcoma	1 (5.1)
Synovial sarcoma	1 (5.1)
Tumour location	*n* (%)
Distal femur	9 (47.4)
Proximal tibia	2 (10.5)
Pelvis	2 (10.5)
Proximal fibula	1 (5.3)
Distal fibula	1 (5.3)
Other	4 (21.0)
Staging	*n* (%)
Presence of metastatic disease	7 (36.8)
Localised disease only	12 (63.2)

Abbreviation: SD, standard deviation.

### 3.2 Reliability

Inter‐rater reliability ICC ranged from 0.91 to 0.99 (excellent reliability) for psoas muscle, G muscle, medial gastrocnemius muscle, mid‐femoral muscle circumference and mid‐tibial muscle circumference. Inter‐rater reliability ICC were 0.83 and 0.88 (good reliability) for BF and RF muscles, respectively. Intra‐rater reliability ICC for all muscles and muscle groups was between 0.92 and 0.99 (excellent reliability), see Table 3.

**TABLE 3:** Inter‐ and intra‐rater reliability and minimal detectable change.

Measurement	Inter‐rater		Intra‐rater		MDC (mm^2^)	MDC as percentage of muscle size (%)
	ICC	95% confidence interval	ICC	95% confidence interval		
Psoas	0.97	0.95–0.98	0.99	0.98–0.99	143.53	18.26
Rectus femoris	0.88	0.83–0.95	0.98	0.97–0.99	114.01	28.55
Gracilis	0.96	0.93–0.98	0.99	0.98–0.99	61.72	27.34
Biceps femoris	0.83	0.75–0.89	0.92	0.87–0.95	285.66	34.77
Medial head gastrocnemius	0.91	0.86–0.94	0.98	0.99–0.99	257.08	39.47
Mid‐femoral muscle circumference	0.99	0.99–0.99	0.99	0.96–0.99	851.85	10.62
Mid‐tibial muscle circumference	0.99	0.99–0.99	0.99	0.99–0.99	528.08	11.69

Abbreviations: ICC, intraclass correlation coefficient; MDC, minimal detectable change.

### 3.3 Minimal Detectable Change

MDC was calculated for each muscle/muscle group and expressed as a percentage of the overall size of the muscle (Table 3).

### 3.4 Range of Measurements

**FIGURE 3:**
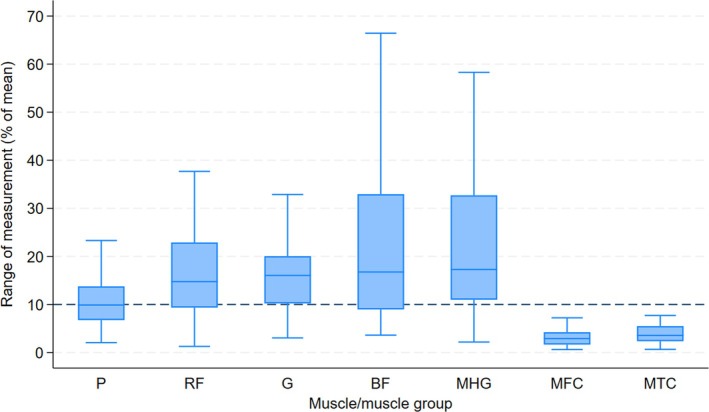
Range of measurements expressed as a percentage of the mean muscle area. The box represents the interquartile range (25th–75th percentiles); the line inside the box indicates the median, and whiskers represent the range excluding outliers. Dotted line marks 10% of the range of measurements. B, Biceps femoris; G, Gracilis; MFC, Mid‐femoral muscle circumference; MHG, Medial head of gastrocnemius; MTC, Mid‐tibial muscle circumference; P, Psoas; RF, Rectus femoris.

Further subanalysis was conducted, analysing the range of measurements for each muscle/muscle group and describing the range as a percentage of the mean area of the muscle (Figure 3).

## 4 Discussion

This is the first study to report the reliability of measuring muscle cross‐sectional area in children with musculoskeletal sarcoma. This study found excellent inter‐ and intra‐observer reliability in measuring the psoas, G, medial gastrocnemius, MFC and MTC. Good reliability was found for the RF and BF muscles. Importantly, this study describes the MDC and the percentage of change that is likely to be beyond measurement error when using this technique. The measurements of psoas, MFC and MTC demonstrate a low MDC related to the size of the muscle, and these muscles therefore have considerable potential as a measurement of change in muscle mass. The results of this study are an important step towards exploring changes in muscle cross‐sectional area over time. By establishing a reliable measurement technique, children with significant muscle loss may be identified, and targeted prehabilitation, nutritional intervention and or physiotherapy/physical therapy intervention can be implemented or enhanced to mitigate the likely poorer physical outcomes.

Measuring muscle volume using segmentation of the whole muscle is considered the gold standard. However, this method requires a sophisticated image analysis. In contrast, single‐slice CT or MRI measurements of muscle cross‐sectional area are an easily accessible and quick measure for clinical practice to assess muscle mass or sarcopenia [23]. Single‐slice cross‐sectional area has been shown to have a high correlation to total muscle volume [28]. Strandberg et al. reported excellent overall reliability of thigh muscle cross‐sectional area in adults undergoing anterior cruciate ligament rehabilitation [17], which is in agreement with the findings of our study. Wang et al. found similarly excellent reliability when assessing interobserver reliability of MRI measurements of abdominal skeletal muscle in adults with kidney disease [16]. There is very limited evidence for the appropriateness of MRI image interpretation by clinicians who are not trained in medical imaging. However, it is generally accepted that increasing experience and training in MRI image interpretation is associated with increased accuracy of findings [29]. For this novel research, the authors have chosen to explore the reliability of using less experienced clinicians who are specifically trained to identify specific muscles in specific MR images after focused training by a specialist radiologist, with the intention of validating the technique for clinical implementation.

By investigating the reliability of measuring muscle area in several muscles and muscle groups in the lower limb, our study supports the use of this technique to interpret muscle area in children undergoing treatment for musculoskeletal sarcoma. The assessment of psoas muscle area in children has gained prominence as a method for evaluating muscle mass and identifying sarcopenia in paediatric populations [30]. Single‐slice CT and MRI imaging techniques are commonly employed to measure psoas muscle area at specific lumbar levels, notably L3–4 and L4–5. A study by Lurz et al. [22] established age‐ and sex‐specific reference values for total psoas muscle area at these intervertebral levels in children aged 1 to 16 years, providing a foundation for identifying sarcopenia in paediatric patients. The ICCs for the psoas muscle area reported by Lurz et al. (2020) were similar to those in children with musculoskeletal sarcoma reported in our study. The data presented in our study aim to further describe the reliability of this measurement by quantifying the MDC and the range of measurement beyond measurement or random error.

Previous studies have described changes in muscle size during cancer treatment and how they relate to functional and other outcomes [31, 32]; however, these studies did not include musculoskeletal sarcoma. It is important to consider the impact of the significant rehabilitation needs of children who are undergoing treatment for musculoskeletal sarcoma, most importantly the surgical resection of the muscles surrounding the tumour. The relationship between changes in muscle volume and force generation and their relationship to disease, injury or disuse has been described extensively [33, 34, 35, 36]. Assessing muscle size, particularly of the calf and quadriceps, is highly relevant for predicting physical and rehabilitation outcomes in paediatric populations. In children with cerebral palsy, studies have shown that calf muscle volume increases linearly with age but at a slower rate compared with typically developing children [37]. A study involving children with spastic diplegic cerebral palsy found that quantifying leg muscle strength is essential for identifying muscle groups for targeted interventions [38]. Although measurements of muscle strength and size are increasingly used to evaluate musculoskeletal changes and rehabilitation potential in other patient populations, it has yet to be tested in children undergoing treatment for musculoskeletal sarcoma—an important direction for future research.

For children diagnosed with musculoskeletal sarcoma, chemotherapy results in significant deconditioning and changes in musculoskeletal health [4, 39]. In addition to this, children with musculoskeletal sarcoma are also affected by restrictions of weightbearing in the presurgical phase of treatment. Establishing a method for assessment and quantification of these musculoskeletal changes will aid clinicians in identifying children at greater risk of poor outcomes after surgery, enable the allocation of resources and guide prehabilitation. Two methods can be utilised to do this. Firstly, comparison can be made to sex and age related normative data provided by Lurz et al. [22]. This requires the clinician to have confidence in the reliability of the measurement. The new data provided in this study support the use of the cross‐sectional area of muscle as a reliable measurement in children with musculoskeletal sarcoma. Secondly, the comparison of muscle mass can be completed in a single child over time. This is particularly pertinent when children are receiving multiple sequential MRI or CT scans as is the case in routine treatment for musculoskeletal sarcoma. By calculating the MDC, this study aims to allow future researchers to determine whether changes in muscle cross‐sectional area are considered outside the estimated measurement error. The results across the different muscles/muscle groups vary in size, which reflects the size of the muscle as well as the reliability of the measurement. Previous studies have attempted to explore what counts as meaningful change in muscle mass in adults [40]; however, this has not been undertaken in children. To describe the variation between examiners in each measurement, we calculated the mean of the measurements of each muscle/muscle group and expressed the range of those measurements as a percentage of the mean (Figure 3). This allowed identification of a percentage change that is considered likely (above 95%) to be above the margin of measurement error or random variation. The data presented in our study support clinicians to identify the reliability of the measurement. For example, a change of greater than 10% of muscle area in the MFC or MTC can be confidently attributed to be outside of the measurement error.

Using MRI or CT scanning to identify children with significant muscle loss is a simple and reliable technique that can be utilised in the clinical arena. Children undergoing treatment for sarcoma receive sequential MRI or CT imaging as part of standard care. Therefore, this non‐invasive method can be implemented without significant cost or additional burden to children or families.

This study has several limitations, including acquiring accurate images during MRI, manual methodologies and assessment of other tissue components beside skeletal muscles. Maintaining a stationary position during MRI scans can present an additional challenge within the paediatric setting. Furthermore, muscle or joint contractures may affect positioning within the machine and subsequently influence muscle area measurements. Manual segmentation and measurement of muscle were conducted for this study; however, future methodologies may integrate some degree of automation into this process. This study did not seek to examine the effects of fat infiltration into muscle tissue, which may signify a missed opportunity to quantify other facets of muscle quality. This study did not explore differences in prepubertal and postpubertal children. Future studies should consider this factor when conducting any subanalysis.

The data presented in this study indicate that MRI measurement of muscle cross‐sectional area in children and adolescents undergoing treatment for musculoskeletal sarcoma is a reliable tool that does not impose an additional burden on clinical care. Utilising this measurement during the presurgery stage of treatment may allow clinicians to identify children experiencing significant deconditioning and facilitate early intervention prior to definitive surgery to enhance physical outcomes postoperatively.

## Conflicts of Interest

The authors declare no conflicts of interest.
